# Investigation of Scribing Parameters’ Influence on the Tomography and Crack Initiation of OLED Display Panels for Circular Structures

**DOI:** 10.3390/mi16091071

**Published:** 2025-09-22

**Authors:** Huaye Kong, Xijing Zhu, Guohong Li, Yao Liu

**Affiliations:** School of Mechanical Engineering, North University of China, Taiyuan 030051, China

**Keywords:** OLED display panel, scoring-wheel cutting, crack propagation, Taguchi experiment, parameter optimization, morphological characteristics

## Abstract

This paper focuses on the scoring-wheel cutting process for circular structures of OLED display panels, conducting in-depth research through an experiment–analysis–optimization system. Based on the Taguchi experimental design, a three-factor, five-level experiment is conducted, with the blade wheel angle (A), cutting speed (B), and pressure (C) set as influencing factors, and the scratch depth (h), width (w), median crack depth (l), and transverse crack width (d) set as evaluation indicators. The experiments are carried out using a self-developed dicing-wheel cutting device, and the morphology, roughness, and hardness of the cutting surface and cross-section are characterized by means of ultra-depth-of-field microscopy, laser confocal microscopy, microhardness tester, and other equipment. The research shows that the order of factors affecting the cutting quality is as follows: A > C > B. Through the analysis of morphology and crack characteristics, it is determined that the optimal parameter combination is a dicing wheel angle of 130°, a cutting speed of 20 mm/s, and a pressure of 11 N. The verification results indicate that this combination can reduce surface roughness, stabilize hardness, and realize efficient and precise processing of special-shaped structures in OLED display panels, providing strong theoretical and technical support for industrial process optimization.

## 1. Introduction

OLED display panels, with their prominent advantages such as self-luminescence, high contrast, thinness, lightness, and bendability [1,2], have become indispensable core components in fields like smart terminals and wearable devices. As the market demand for personalized products such as curved screens and foldable screens continues to rise, the high-precision processing of special-shaped structures [3,4] (such as small holes, curved edges, and non-right-angle corners) has become a key link determining product yield and performance in the OLED panel manufacturing process. Among the current cutting technologies, scoring-wheel cutting technology [5] is widely used in the processing of special-shaped structures on the glass substrates of OLED display panels due to its characteristics of low cost, high efficiency, and strong adaptability.

**Figure 1:**
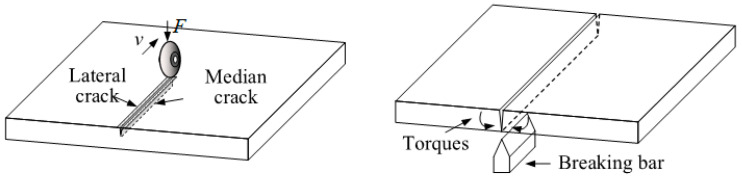
Schematic diagram of the cutting process.

Scoring-wheel cutting forms scratches on the glass surface through mechanical force and induces median cracks to propagate along a preset path, ultimately achieving material separation [6,7]. Figure 1 shows a schematic diagram of this process [8,9]. During the scoring-wheel cutting process, the cemented carbide wheel directly exerts stress on the surface of brittle materials, leading to the formation of surface microcracks and subsurface damage layers. The cutting quality is easily affected by the coupling of process parameters such as cutter wheel angle (A), cutting speed (B) and pressure (C), as well as material properties. Insufficient precision can easily cause crack deviation, edge chipping, transverse cracks, etc. [10,11]. For substrates with a thickness of ≤0.5 mm especially, slight fluctuations in parameters may cause damage to the functional layer, directly affecting product yield.

In the face of these urgent cutting quality challenges, scholars at home and abroad have made significant achievements in improving cutting process routes and optimizing key process parameters. Numerous researchers have conducted in-depth studies on the effects of factors such as cutting-wheel-related parameters, cutting speed, and load on glass cutting quality. Kim et al. [12] optimized the cutting speed and load parameters through orthogonal experiments, reducing the roughness of the cutting edge by 30%. Cheng et al. [13] found that ultra-thin glass processed by mechanical cutting exhibits obvious edge chipping and sidewall damage, which leads to a significant reduction in its bending strength. Liu et al. [14] optimized process parameters such as the scoring wheel angle, the scoring force, and the penetration depth. This optimization reduced the amount of glass debris generated on the surface of the glass substrate after cutting/fracturing and lowered the content of glass debris contaminants in the subsequent polarizer lamination process by 1% to 2%. Pei et al. [15] conducted an in-depth exploration of the influence law of different parameters on longitudinal cutting marks. By comprehensively considering three key parameters—dicing wheel angle, tool depth, and pressure—they ultimately achieved better cutting performance. Luo et al. [16] studied the characteristics and wear patterns of thin diamond dicing wheels in the cutting and grinding of BK7 optical glass; they discovered that reducing the transverse speed can result in better cutting straightness. Li et al. [17] systematically sorted out the influencing factors of defect formation and carried out experimental verification, which significantly improved the cutting quality and yield rate.

Other scholars have also conducted research on the cracks generated by factors such as cutting speed and load during the glass-cutting process. Swain et al. [18] used indentation fracture mechanics to determine the impact of load and tool geometry on the depth of median cracks generated during glass cutting. Pan et al. [19] studied the effect of different cutting pressures and cutter wheel depths on the size of median cracks and transverse cracks, and found that increasing the cutting pressure would lead to an increase in the length of median cracks and transverse cracks. Liu et al. [20] analyzed the characteristics such as the direction, length, and depth of the cracks, and successfully established an empirical formula between crack depth and load through the accumulation and induction of a large amount of experimental data, which can relatively accurately predict the crack depth. Ono et al. [21] explored the influence of cutter wheel angle and diameter on the scribing and fracture of liquid crystal display glass substrates. The results showed that, when a cutter wheel with an angle of 130° and a diameter of 4 mm was used, the required fracture force was the smallest and there was no transverse crack propagation, which was the optimal result for glass substrate separation. Hasegawa et al. [22] found that, when a 2.0 mm serrated diamond wheel scribes 0.7 mm non-alkali glass, a load of 9–10 N is the critical point, and the crack-propagation behavior and fracture surface morphology differ significantly before and after this point. Zhang et al. [23] found that, when a cutting wheel with a cutting angle of 60–80° is used for cutting, the propagation of transverse cracks in the glass substrate is significantly suppressed. Murakami et al. [24] demonstrated that, when there is no constraint at the bottom, the contact force acting on the crack surface not only promotes crack propagation but also causes the matrix to bend, and the deformation at the bottom has a significant impact on the formation of secondary cracks. Li [25] found that the scratch formation mechanism of ultra-thin glass was optimal when the knife wheel angle was 120°, the scratching force was 20 N, and the scratching speed was 100–400 mm/s, without any damage or peeling.

However, current research on scoring-wheel cutting of OLED display panels mostly focuses on symmetric and simple structures, while studies on the cutting mechanism and parameter control for complex special-shaped structures such as curved edges and non-right-angle corners remain insufficient. This research takes the scoring-wheel cutting of special-shaped structures (e.g., circular trajectories) on OLED display panels as the research object, and systematically analyzes the influence law of process parameters on cutting quality and crack-propagation behavior during the scoring-wheel cutting process through an “experiment–analysis–optimization” research model. It aims to provide a theoretical basis and technical support for the efficient and precise processing of OLED display panels with special-shaped structures.

## 2. Experimental Design and Setup

### 2.1. Experimental Design Method

The Taguchi experimental approach is an efficient quality-improvement method [26]. In this study, Minitab17 was used to design experiments and analyze data. The signal-to-noise ratio (SN ratio) was employed to measure the robustness of quality characteristics and reduce process fluctuations. Three factors, namely cutter wheel angle A (100°, 110°, 130°, 140°), cutting speed B (10 mm/s, 15 mm/s, 20 mm/s, 25 mm/s, 30 mm/s), and scribing load C (9 N, 11 N, 13 N, 15 N, 17 N), were selected as influencing factors. The evaluation indices included the cutter wheel groove depth, h, the cutter wheel groove width, w, the median crack depth, l, and the lateral crack, d. A three-factor, five-level experiment was designed. Table 1 and Table 2 show the table of the experimental factors and levels and the Taguchi experimental design table, respectively.

### 2.2. Experimental Equipment and Materials

#### 2.2.1. Construction of Scoring-Wheel Cutting Experimental Platform

**Figure 2:**
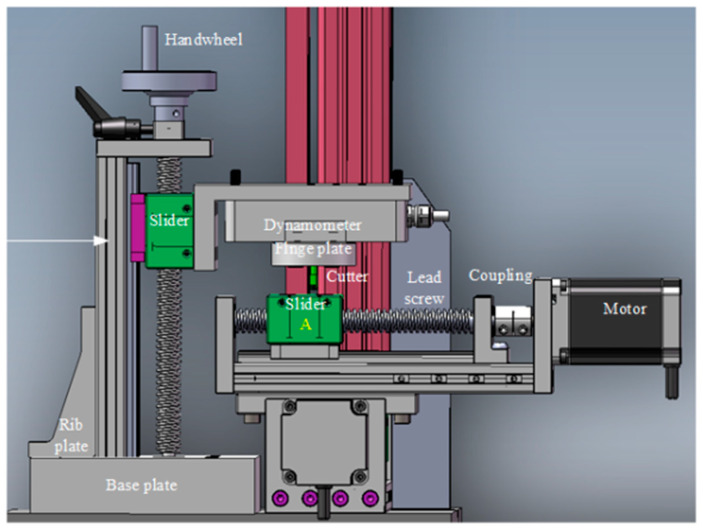
Three-dimensional diagram of the experimental device.

In this study, a self-developed scoring-wheel cutting device was used for glass cutting (Figure 2 shows the 3D diagram of the device). The cutter wheel is made of cemented carbide, and the precision of the drive system is 0.05 mm. During the calibration of the device, a glass with alignment marks is taken, and the alignment point is aligned with the tip of the cutter. Then, the motor is controlled to drive the alignment glass to move so that the alignment point is aligned with the laser indicator point. The XY axis coordinates of the panel during the two alignments are recorded, and the coordinate difference between the starting point of the cutter wheel and the initial laser point is obtained by subtracting these coordinates, which is then written into the controller. The glass is then fixed on the surface of slider A of the horizontal guide rail, and the metal cutter wheel is positioned above the glass, which can move up and down via the vertical guide rail. The two-dimensional moving platform drives the glass to move, enabling the cutter wheel to inscribe the expected special-shaped grooves onto the glass surface.

The characterization of the microstructure and properties involved in the experiment mainly includes observing the surface and cross-sectional morphology of the display panel after the scoring-wheel cutting process, measuring the scratch width and depth, the median crack depth, the transverse crack length, the hardness, and the roughness. The main equipment and their models used are listed in Table 3.

#### 2.2.2. Experimental Materials

**Figure 3:**
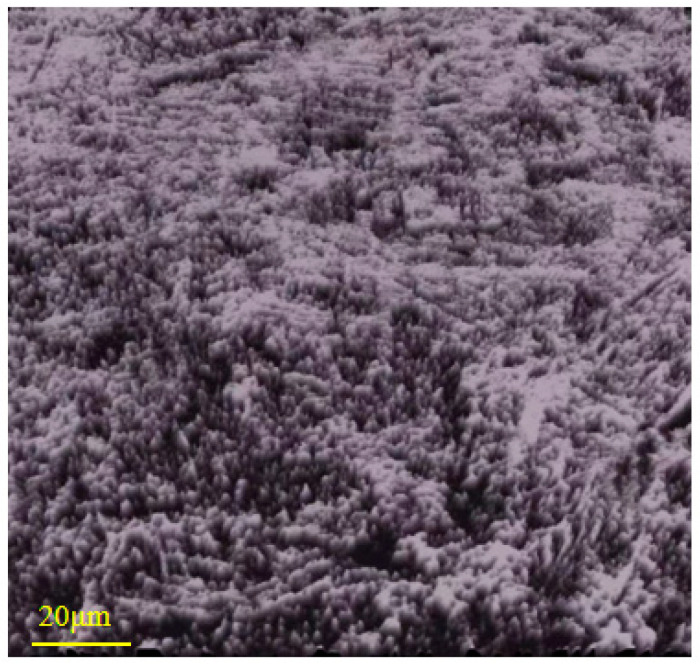
Morphology of soda–lime glass.

In this experiment, a float soda–lime glass with a thickness of 0.5 mm (provided by Luoyang Shangzhuo Technology Co., Ltd., Luoyang, China) was used as the display panel substrate. The surface morphology of the glass magnified 20 times, captured by a confocal microscope, is shown in Figure 3. The elemental composition and content of the soda–lime glass are listed in Table 4.

### 2.3. Preparation of Display Panel Samples

**Figure 4:**
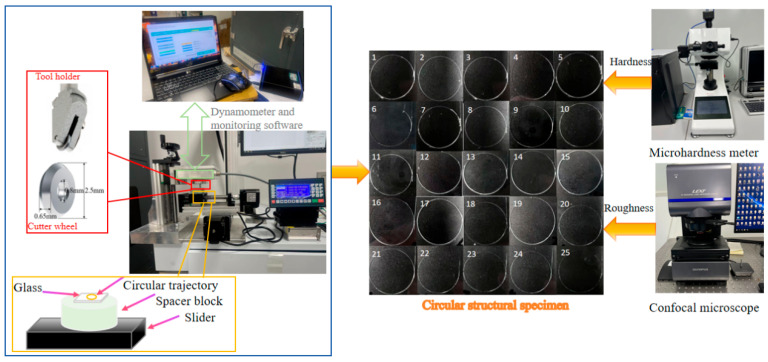
Preparation process of circular structure sample.

The soda–lime glass substrates, with dimensions of 30 mm × 30 mm × 0.5 mm, were cleaned with ethanol, dried, and then fixed on a thick glass backing plate. Using the scoring-wheel cutting device, circular trajectories with a diameter of 20 mm were cut on the substrate surface according to the parameter combinations designed in Table 2 (the trajectory diameter was selected based on project requirements, matching the 20 mm diameter openings commonly used for hardware such as vehicle rearview cameras and biometric modules in smart wearable devices). The process diagram is shown in Figure 4.

### 2.4. Surface Quality Characterization

#### 2.4.1. Morphology Characterization

**Figure 5:**
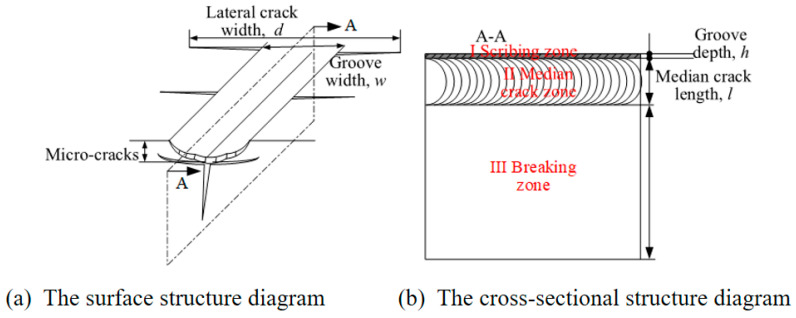
Crack-propagation characteristics of surface and section during blade wheel carving.

The cut samples were ultrasonically cleaned to remove debris, and the microscopic morphology of the scratches was observed using an ultra-depth-of-field microscope. Figure 5 shows the characteristics of surface and cross-sectional crack propagation during cutter wheel scratching. In the surface structure diagram (Figure 5a), the lateral crack width is denoted by *d*, the groove width is marked as *w*, and the distribution of microcracks reflects the mesoscale damage. In the cross-sectional structure diagram (Figure 5b), three regions can be identified: I (scribing zone), II (median crack zone), and III (fracture zone). The groove depth is represented by *h*, and the median crack length is labeled as *l*. During measurement, three different positions were selected for each index of each sample, and the average value was taken.

#### 2.4.2. Roughness Characterization

An unprocessed original glass sample with dimensions of 30 mm × 20 mm was selected as the control sample, while a glass sample of the same material and specification, which had been subjected to scoring-wheel cutting with optimal parameters, was used as the experimental sample. A laser confocal microscope (OLS5000) was employed to collect 3D morphology data and extract line roughness. The measurement evaluation length was set to 1280 µm. The evaluation indices included arithmetic mean roughness (Ra) and root mean square roughness (Rq). During measurement, the samples were fixed on the stage; for each sample, 5 positions were selected, and each position was measured 3 times. The average value of these measurements was calculated, and then the mean value and standard deviation of each index were computed and recorded.

#### 2.4.3. Hardness Characterization

In this study, a Vickers hardness tester was used to measure the cross-sectional hardness of the display panel substrate after cutter wheel scratching. The test parameters were set as a load of 200 gf and a dwell time of 15 s. A laser confocal microscope (at 200× magnification) was used to observe the indentations for evaluating surface integrity, ensuring the absence of millimeter-scale cracks. With the hardness of 570 N/mm^2^ measured from the blank control group as the reference, seven groups of sampling points (1–7 mm) were set at every 1 mm along the cutting line trajectory to analyze the hardness gradient. Additionally, five groups of sampling points (from the surface layer to the center) were arranged at every 0.08 mm along the cross-sectional thickness. Each point was measured three times, and the average value was taken, with the error controlled within 5%.

## 3. Experimental Results and Analysis

In this experiment, with cutter wheel angle (A), cutting speed (B), and pressure (C) as variables, the groove depth, *h*, groove width, *w*, median crack depth, *l*, and lateral crack, *d*, were measured. The experimental results are listed in Table 5. Based on these data, Taguchi analysis was conducted with the signal-to-noise ratio as the core index. Through response table analysis, variance analysis, and model coefficient estimation, the sensitive factors affecting cutting quality were explored, so as to provide support for decision making surrounding the optimal parameters.

### 3.1. Analysis of Signal-to-Noise Ratio Response Table

For scoring-wheel cutting, the smaller the transverse crack length, median crack depth, cutter wheel scratch width, and scratch depth, the better. Therefore, the “smaller-the-better” characteristic is selected for the signal-to-noise ratio (SNR). The calculation formula for the SNR of the smaller-the-better characteristic is as shown in Equation (1) [27]:

(1) $$\mathrm{SNR}=-10\log\left(\frac{1}{n}\sum_{i=1}^{n}{y_{i}}^{2}\right)$$

where y_i_ is the characteristic value and n is the number of repetitions.

It can be seen from the signal-to-noise ratio response table under the smaller-the-better characteristic in Table 6 that A has the largest Delta value and ranks 1, exerting the most significant influence on the SNR, with levels 3 and 4 showing the best robustness. B has a Delta value of 1.28, ranks 3, and has the weakest influence, with small differences in SNR among various levels. C ranks 2 in terms of Delta value, and the SNR values at levels 4 and 3 are relatively better.

**Figure 6:**
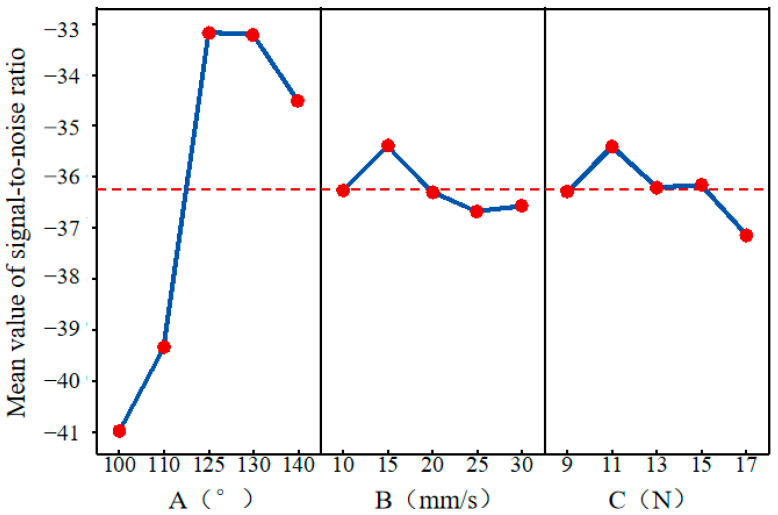
The main effect diagram of the signal-to-noise ratio.

The main effect plot of the signal-to-noise ratio (Figure 6) intuitively presents the relevant trends: the SNR reaches its peak when the A is in the range of 125–130°; the SNR performs well when the B is at a medium–low level of 10–20 mm/s and the C is in the range of 11–15 N.

### 3.2. Analysis of Variance for Signal-to-Noise Ratio

The results of the analysis of variance (ANOVA) for the signal-to-noise ratio are shown in Table 7, which presents the ANOVA results with the signal-to-noise ratio as the response variable and A, B, and C as influencing factors. This analysis can be used to test the contribution of each factor to the variation in cutting quality stability and their statistical significance.

**Figure 7:**
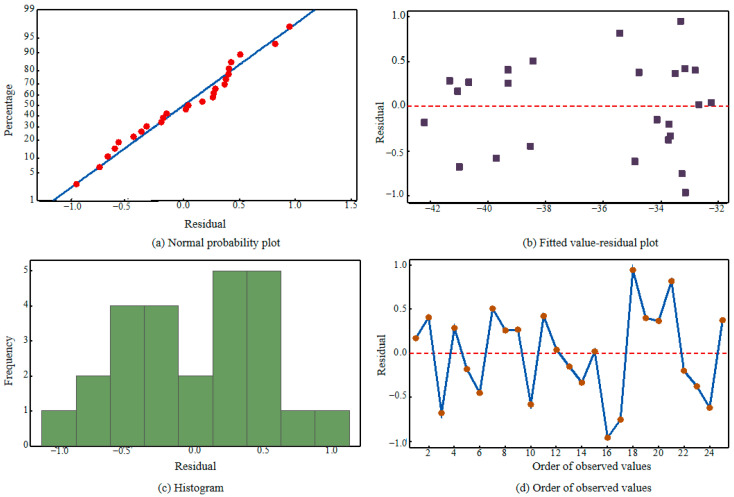
Ratio residual diagrams of the signal-to-noise ratio. (**a**) Normal probability plot; (**b**) Fitted value-residual plot; (**c**) Histogram; (**d**) Order of observed values.

The data in Table 7 show that A contributes the most to the variation of the signal-to-noise ratio (93.5%), with high Adj MS and F values, and *p* = 0.000 < 0.05, making it an extremely significant core factor. C contributes 2.6%, with F = 3.73 and *p* = 0.034 < 0.05, which is a significant secondary factor. B contributes 1.8%, with F = 2.51 and *p* = 0.098 > 0.05, indicating an insignificant influence. The residual error accounts for 2.1%, showing that the model has small errors and strong explanatory power. Combined with the residual plot in Figure 7, the model fits well. The priority order is A > C > B, so the cutter wheel angle should be adjusted first during optimization.

### 3.3. Estimation of Signal-to-Noise Ratio Model Coefficients

A linear model analysis was performed on the signal-to-noise ratio with respect to the cutter wheel angle, cutting speed, and pressure, and the results of the model coefficient estimation are shown in Table 8. The model [28] is generally expressed as:

(2) $$SN=\beta_{0}+\beta_{A1}A_{1}+\beta_{A2}A_{2}+\beta_{B1}B_{1}+\ldots$$

where β_0_ is a constant (intercept), representing the mean SNR when all factors are at the reference level (e.g., A1, B1); β_A2_ is the effect value of factor A at level 2 (the increment of SNR relative to the reference level A1); the other coefficients follow the same logic. A positive value indicates that the level is better than the reference level, and the larger the absolute value, the stronger the effect.

According to Expression (2), the model expression can be derived as follows (3):

(3) $$\begin{array}{c} \mathrm{SN}=-36.2391-4.7371A_{1}-3.0895A_{2}+3.0613A_{3}+3.0273A_{4}-0.0199B_{1}+0.8436B_{2}-0.0594B_{3}-0.4346B_{4}-0.0508C_{1}\;+\\ 0.8323C_{2}+0.0381C_{3}+0.0791C_{4}+\ldots \end{array}$$

Table 8 shows that the cutter wheel angles of 100° and 110° significantly reduce the signal-to-noise ratio, while 125° and 130° significantly increase it. For all angles, *p* = 0.000 < 0.05, and the absolute values of T-values range from 0.644 to 16.656, indicating an extremely significant influence. A cutting speed of 15 mm/s slightly increases the signal-to-noise ratio (*p* = 0.012), while other speeds have no significant impact. A pressure of 11 N slightly increases the signal-to-noise ratio relative to the reference level, and the effects of other pressures are negligible. The model has S = 0.7110 and R-Sq = 97.9%, indicating a high degree of fitting and a good ability to explain the changes in the signal-to-noise ratio.

Based on the comprehensive analysis of the Taguchi experiment on the signal-to-noise ratio, the cutter wheel angle is the key factor affecting the cutting quality. When the angle is in the range of 125–130°, the signal-to-noise ratio is the highest, corresponding to better cutting quality and stronger stability. A pressure of 11 N can improve robustness and reduce fluctuations. The influence of cutting speed is relatively weak, and it has a better auxiliary optimization effect when ranging from 10–20 mm/s. The residual plots conform to the normal distribution, verifying that all models have a reasonable degree of fit.

## 4. Determination of Parameter Combination Based on Morphological Characteristics

### 4.1. Determination of Cutter Wheel Angle

**Figure 8:**
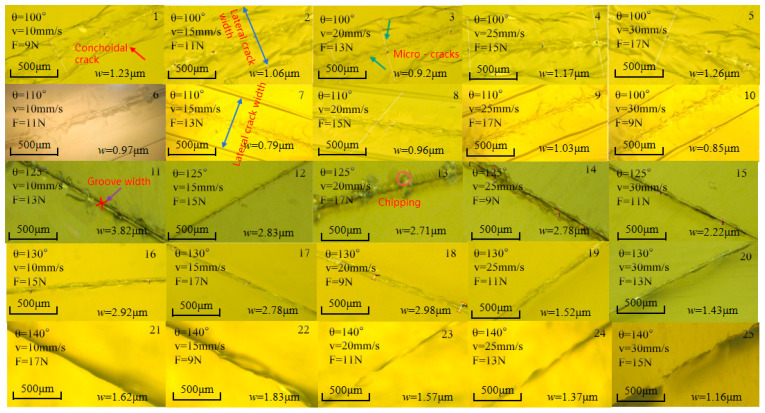
Surface morphology of cutting display panel substrate under different process parameters.

Figure 8 investigates the influence of cutter wheel angle, θ, cutting pressure, F, and speed, v, on the crack morphology of the circular-structured display panel substrate. When θ = 100°, the adaptability between the cutter wheel and the circular trajectory is poor, resulting in uneven force on the glass. The scratches show a shell-like shape, with transverse cracks ranging from 135.36 to 228.24 µm in length. At θ = 110°, the change in cutter wheel angle adjusts the force on the glass, and the width of the main scratch stabilizes at 0.79–1.03 µm. However, the circular trajectory still causes the propagation of small cracks, with transverse cracks ranging from 117.36 to 167.72 µm in width. When θ = 125°, the circular structure leads to a wide distribution of transverse stress, resulting in a “plowing phenomenon”, with long damage zones accompanied by chipping. At θ = 130°, the cutter wheel has good adaptability to the circular structure, ensuring uniform force. The cutting marks are uniform at 2.43–2.92 µm, and cracks are suppressed to 8.23–9.87 µm, achieving precise cutting. When θ = 140°, the angle is incompatible, and the circular trajectory amplifies stress fluctuations, leading to unstable cutting. The scratch width ranges from 3.12 to 3.66 µm, and the crack width ranges from 14.57 to 18.08 µm. As the angle increases, the scratch width also increases. However, this width is still far smaller than the minimum design tolerance of the OLED circular functional area (usually ≥5 µm) and does not exceed the coverage capability of subsequent packaging processes (10–20 µm). In summary, the cutter wheel angle has a significant impact on cutting quality, with 130° being optimal, which is consistent with the conclusions of the Taguchi experiment.

### 4.2. Determination of Cutting Speed

To determine the optimal cutting speed, based on the conclusions in Section 3, the cutter wheel angle was set to 130° and the cutting pressure was set to 11 N. The single-factor method [29] was used to study the influence of cutting speed on the cutting quality parameters of the display panel substrate, including the transverse crack width, d, the cutting width, w, the depth, h, and the median crack depth, l. In the experiment, six cutting speed levels were set in the range of 10–20 mm/s with a gradient of 2 mm/s. To ensure the statistical significance of the experimental data, all experimental groups were repeated three times, and the average value of each parameter combination after three repeated experiments was taken as the experimental result. The experimental design table and the results are shown in Table 9.

#### 4.2.1. Influence of Cutting Speed on Cutting Surface Morphology and Transverse Cracks

**Figure 9:**
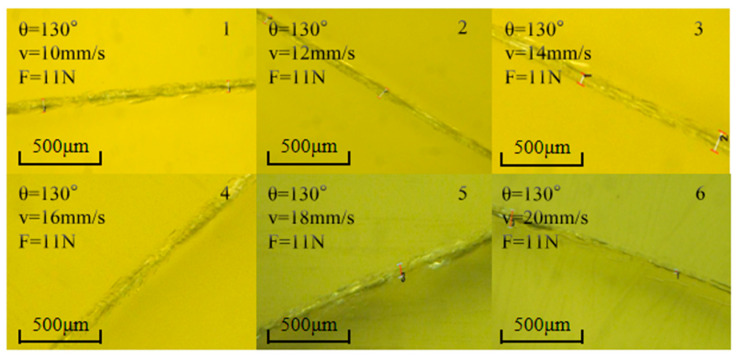
θ = 130°, F = 11 N, surface morphology at different cutting speeds.

Figure 9 shows the influence of cutting speed in the range of 10–20 mm/s on crack morphology under the conditions of a cutter wheel angle of 130° and a pressure of 11 N: an increase in cutting speed leads to a gradual decrease in the width of cutting marks by 23%, with no obvious damage to surface integrity. The length of transverse cracks decreases by 16%, but there is no significant change in their morphology. This indicates that, within the experimental range, the cutting speed has little impact on the cutting surface morphology, which verifies the conclusion from the Taguchi experiment that the influence of cutting speed is relatively weak.

**Figure 10:**
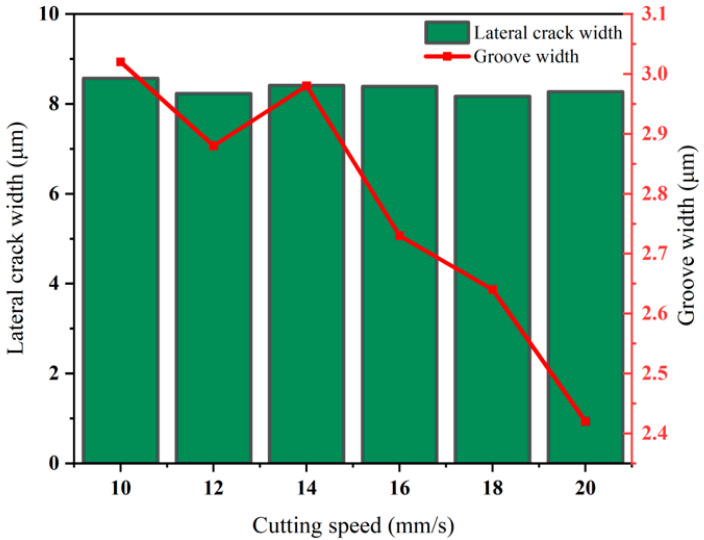
Lateral crack length and blade wheel scratch width at different cutting speeds.

Figure 10 shows the influence of different cutting speeds on the transverse crack width and the cutter wheel scratch width: due to the good adaptability of the 130° cutter wheel, the transverse crack width is stable at 8–9 µm; at 10–12 mm/s, the tool moves more smoothly along the curved trajectory with uniform contact, and the scratch width decreases from 3.05 µm to 2.9 µm; at 14 mm/s, the inertia of the cutter wheel superimposed with the trajectory change causes vibration, resulting in uneven energy distribution and stress concentration, thus widening the scratch; at 14–20 mm/s, the cutter wheel “sweeps” quickly, shortening the action time on the curved trajectory and reducing extrusion deformation. Moreover, the 130° cutter wheel maintains stable stress release, and the scratch width decreases to 2.3 µm. Considering the goals of controlling cracks or pursuing narrower scratches, the optimal cutting speed is selected as 20 mm/s.

#### 4.2.2. Influence of Cutting Speed on Cross-Sectional Morphology and Median Cracks

**Figure 11:**
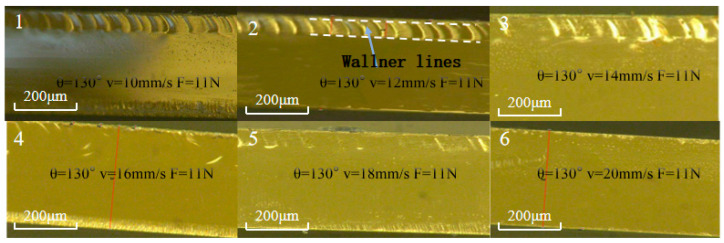
The cross-sectional morphology of cutting display panel substrates under different process parameters.

Figure 11 shows the influence of varying cutting speeds in the range of 10–20 mm/s on the cutting cross-section quality under the conditions of a cutter wheel angle of 130° and a cutting pressure of 11 N in the single-factor experiment. The results indicate that changes in cutting speed have no significant impact on the cross-section quality, suggesting that the parameters affecting cross-section quality are mainly the cutter wheel angle and cutting pressure.

**Figure 12:**
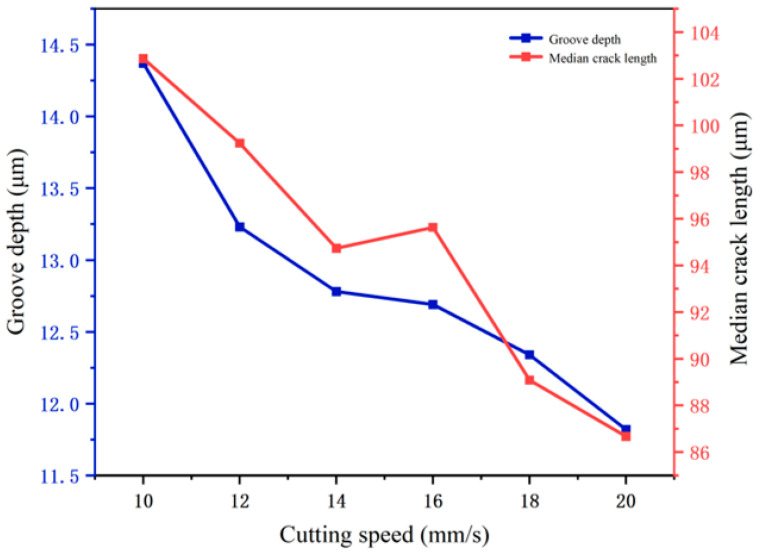
Influence of cutting speed on cutting depth and median crack size.

Figure 12 shows the influence of cutting speed on cutting depth and median crack depth during the cutting of circular structures: Within the range of 10–20 mm/s, both the cutting depth and median crack depth generally decrease as the speed increases, with calculated standard deviations of 1.557 and 1.162, respectively, indicating small fluctuations. Between 14 and 16 mm/s, due to the curved trajectory changing the distribution of cutting heat and stress, the interaction state between the tool and the material is disturbed, resulting in slight fluctuations. For circular structures, a high speed of 20 mm/s enables the cutter wheel to quickly “sweep across” the curved trajectory, shortening the local stress action time, inhibiting the crack propagation in the depth direction, and making both indicators reach relatively low values.

**Figure 13:**
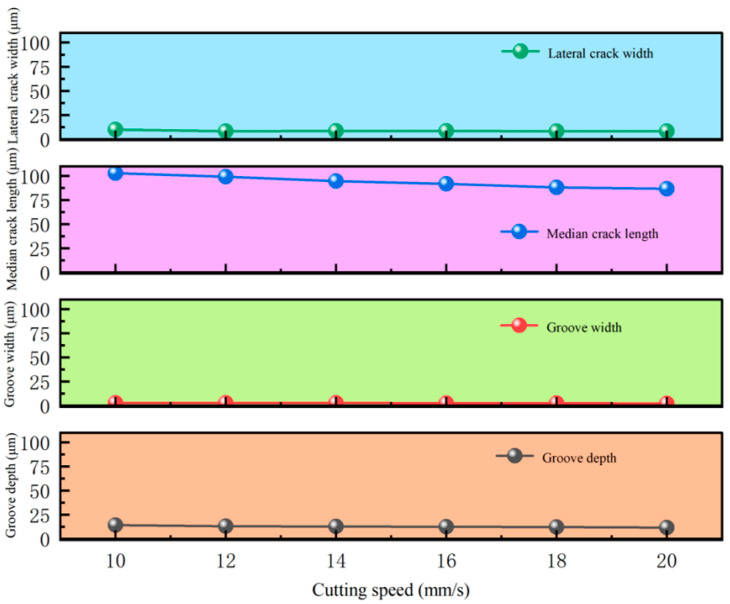
Influence of cutting speed on transverse crack depth, median crack depth, blade wheel scratch width, and blade wheel scratch depth.

Figure 13 presents the influence of cutting speed on transverse crack depth, median crack depth, cutter wheel scratch width, and cutter wheel scratch depth. It can be seen from the figure that cutting speed has almost no impact on transverse crack depth and cutter wheel scratch width, and these two indicators are highly stable. It has a weak inhibitory effect on median crack depth and cutter wheel scratch depth, but the reduction range is extremely small (<5 µm), so the regulatory value of speed on these four indicators can be ignored in actual processes.

Comprehensively considering the influence of cutting speed on surface morphology, transverse cracks, cross-sectional morphology, and median cracks, the following conclusions can be made: under the fixed conditions of a cutter wheel angle of 130° and a pressure of 11 N, the cutting speed within the range of 10–20 mm/s has a weak impact on the cutting quality. However, the performance is optimal at 20 mm/s; with a minimum scratch width of 2.3 µm, the transverse crack length was stably maintained at 8–9 µm and was effectively suppressed; moreover, both the cutter wheel scratch depth (11.8 µm) and the median crack depth (87 µm) reached relatively low values, resulting in better surface and cross-sectional quality. Therefore, the optimal parameter combination for scoring-wheel cutting is as follows: a cutter wheel angle of 130°, a cutting speed of 20 mm/s, and a pressure of 11 N.

## 5. Verification of Optimal Parameter Combination

This section verifies the applicability and advantages of the optimal parameter combination in the glass-cutting process through roughness and hardness tests.

### 5.1. Roughness Analysis

As can be seen from Table 10, which presents the measurement results of the control group and the experimental group, the Ra of the experimental group decreased by 9.5%, indicating an improvement in the average surface smoothness; the Rq decreased by 11.9%, showing that the surface microstructure is more gentle; the Rt decreased by 9.2%, meaning the total drop between peaks and valleys as a whole was reduced. These results indicate that the process effectively optimized the micro-morphology of the glass surface. The combination of a cutter wheel angle of 130°, a pressure of 11 N, and a cutting speed of 20 mm/s, by matching the brittleness of the glass, balancing the pressure, and synergizing with the speed, reduced the incidence of problems such as sharp peaks and deep valleys. The process stability is good and can be used as the preferred parameter for precision glass cutting.

**Figure 14:**
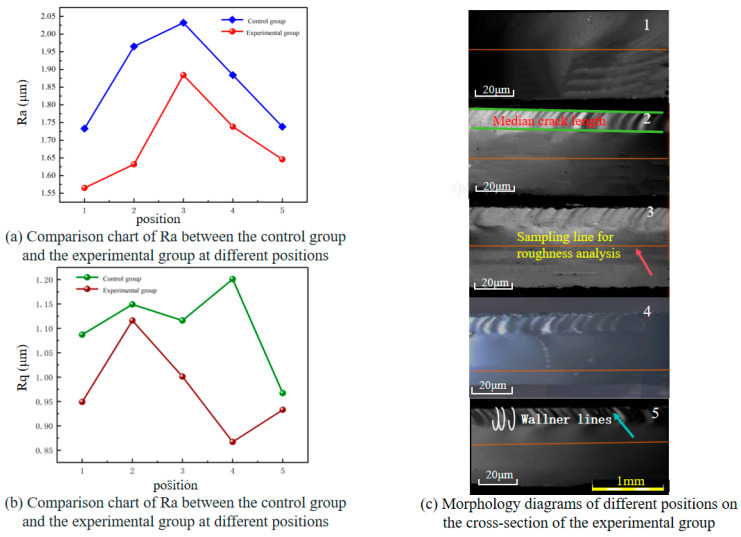
Comparison of Ra and Rq between the control group and the experimental group at different positions and their corresponding cross-sectional morphology diagrams. (**a**) Comparison chart of Ra between the control group and the experimental group at different positions; (**b**) Comparison chart of Ra between the control group and the experimental group at different positions; (**c**) Morphology diagrams of different positions on the cross-section of the experimental group.

As can be seen from Figure 14a,b, the distribution patterns of Ra and Rq differ between the control group and the experimental group. Thanks to its technological advantages, the experimental group effectively suppresses surface damage, with generally lower Ra and Rq values at all positions and gentle fluctuations. Corresponding to the morphology diagrams of the experimental group (Figure 14c), different positions show distinct characteristics under the influence of stress distribution. The overall morphology is consistent with the roughness data, reflecting that the cutting process of the experimental group has better stability, which can maintain relatively uniform and controllable surface quality and reduce the adverse impact of scoring-wheel cutting on cross-sectional roughness.

### 5.2. Hardness Testing

**Figure 15:**
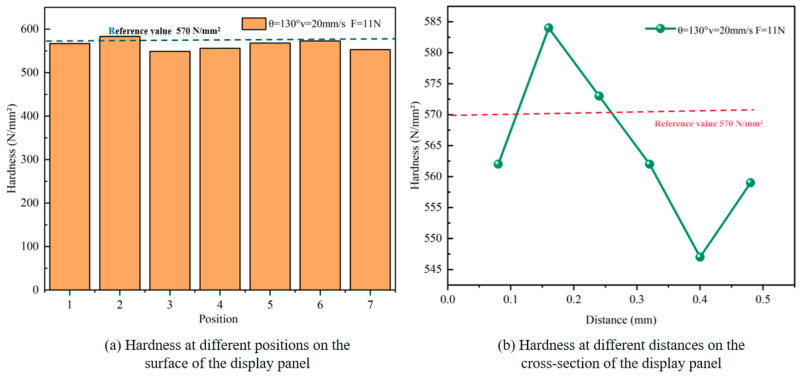
Hardness distribution on the surface and section of circular structure. (**a**) Hardness at different positions on the surface of the display panel; (**b**) Hardness at different distances on the cross-section of the display panel.

Figure 15 presents the surface and cross-sectional hardness characteristics of the circular-structured glass after scoring-wheel cutting under the process parameters of a cutter wheel angle of 130°, a cutting speed of 20 mm/s, and a pressure of 11 N, with a reference hardness value of 570 N/mm^2^.

Figure 15a shows that, when cutting the circular-structured panel, the surface hardness fluctuates slightly around the reference value, with a variation amplitude of approximately 30 N/mm^2^ and low dispersion. This is because the 130° cutter wheel is well-adapted to the circular trajectory, leading to uniform contact areas and stress distribution, which results in highly consistent damage to the glass microstructure at different positions. At a cutting speed of 20 mm/s, the cutter wheel “sweeps” along the circular trajectory, maintaining a stable rhythm of thermal–mechanical interaction, thus ensuring good repeatability of glass deformation and thermal processes. The 11 N pressure guarantees effective indentation while avoiding excessive fragmentation or discontinuity. The synergy of these parameters contributes to the stability of the surface hardness. In Figure 15b, the cross-sectional hardness fluctuates by approximately 26% as the depth increases. The 130° cutter wheel determines the direction of stress and the path of stress transmission. The continuous steering of the circular trajectory causes stress concentration at shallow depths, and the drastic changes in the microstructure lead to a sharp increase in hardness. As the depth increases, the stress disperses and attenuates, resulting in a decrease in hardness. At a cutting speed of 20 mm/s, heat accumulates rapidly at shallow depths to form a hardened layer, while at greater depths, heat diffuses, the force attenuates, and the thermal–mechanical coupling is weak. The 11 N pressure directly squeezes the shallow depths to increase hardness, but at greater depths, the force transmission loss is large and residual stress is released. These factors collectively cause fluctuations in cross-sectional hardness, reflecting the influence of cutting parameters for circular structures on cross-sectional hardness.

## 6. Conclusions

This study focuses on the scoring-wheel cutting process for the special-shaped structures of OLED display panels. Through Taguchi experimental design and morphological characteristic analysis, this study systematically explores the influence of cutter wheel angle, cutting speed, and scribing load on cutting quality. The main conclusions are presented here.

Priority of influencing factors: The cutter wheel angle is the core factor determining cutting quality (contributing 93.5% to the variation of the signal-to-noise ratio, *p* = 0.000), followed by pressure (contributing 2.6%, *p* = 0.034), while the influence of cutting speed is the weakest (contributing 1.8%, *p* = 0.098).

Optimal parameter combination: The optimal parameters are a cutter wheel angle of 130°, a cutting speed of 20 mm/s, and a pressure of 11 N. Under this combination, the cutter wheel has the best adaptability to the circular trajectory with uniform force. The transverse crack width is stable at 8–9 µm, the scratch width is the smallest (2.3 µm), and the median crack depth reaches 87 µm, achieving debris-free precision cutting.

Quality verification results: After cutting with the optimal parameters, the surface roughness Ra is reduced by 9.5% and Rq by 11.9%. The fluctuation of cross-sectional hardness is controlled within a reasonable range, and the surface hardness fluctuates slightly around the reference value (570 N/mm^2^). The overall processing quality and stability are significantly improved.

## Figures and Tables

**Table 1:** Experimental factors and levels.

Levels	Factors		
	A (°)	B (mm/s)	C (N)
1	100	10	9
2	110	15	11
3	125	20	13
4	130	25	15
5	140	30	17

**Table 2:** Experimental design of Tiankou.

Serial Number	A (°)	B (mm/s)	C (N)
1	100	10	9
2	100	15	11
3	100	20	13
4	100	25	15
5	100	30	17
6	110	10	11
7	110	15	13
8	110	20	15
9	110	25	17
10	110	30	9
11	125	10	13
12	125	15	15
13	125	20	17
14	125	25	9
15	125	30	11
16	130	10	15
17	130	15	17
18	130	20	9
19	130	25	11
20	130	30	13
21	140	10	17
22	140	15	9
23	140	20	11
24	140	25	13
25	140	30	15

**Table 3:** List of main equipment and models for the experiment.

Instrument Name	Instrument Model	Manufacturer	City	Country
Ultrasonic cleaner	KQ2200E	Kunshan Ultrasonic Instrument Co., Ltd.	Kunshan	China
Laser confocal microscope	OLS5000	Olympus Corporation	Tokyo	Japan
Touch screen microhardness tester	HVST-1000Z	Shanghai Taiming Optical Instrument Co., Ltd.	Shanghai	China
Super-depth-of-field microscope	VH-Z500R	Keyence Corporation	Osaka	Japan

**Table 4:** Main components and contents of sodium calcium glass.

Component	Fe_2_O_3_	Al_2_O_3_	MgO	CaO	Na_2_O + K_2_O	SiO_2_
Content	0.1 ± 0.02	1 ± 0.5	4 ± 0.5	13.5 ± 0.5	8.1 ± 0.4	72 ± 0.5

**Table 5:** Experimental results data.

Serial Number	A (°)	B (mm/s)	C (N)	*h* (µm)	*w* (µm)	*l* (µm)	*d* (µm)
1	100	10	9	14.38	1.23	136.58	173.52
2	100	15	11	15.57	1.06	111.55	135.36
3	100	20	13	17.43	0.92	128.69	204.84
4	100	25	15	15.87	1.17	118.18	191.52
5	100	30	17	11.58	1.26	130.63	228.24
6	110	10	11	15.52	0.97	98.48	146.88
7	110	15	13	16.12	0.79	104.27	117.36
8	110	20	15	16.63	0.96	107.43	142.56
9	110	25	17	22.73	1.03	123.13	167.72
10	110	30	9	16.64	0.85	125.73	163.36
11	125	10	13	12.75	3.32	85.12	9.56
12	125	15	15	14.34	2.83	80.12	5.28
13	125	20	17	13.67	2.71	102.46	6.34
14	125	25	9	16.31	2.78	98.53	7.61
15	125	30	11	13.48	2.22	84.32	8.73
16	130	10	15	15.37	2.92	99.86	9.87
17	130	15	17	12.93	2.78	99.24	8.23
18	130	20	9	12.38	2.98	81.73	8.41
19	130	25	11	10.06	2.52	82.34	9.35
20	130	30	13	9.58	2.43	89.78	8.78
21	140	10	17	12.62	3.12	105.32	16.75
22	140	15	9	10.67	3.34	97.46	14.57
23	140	20	11	12.85	3.46	99.32	15.87
24	140	25	13	11.71	3.49	117.45	17.32
25	140	30	15	13.61	3.66	102.12	18.08

**Table 6:** Signal-to-noise ratio response table (smaller-the-better).

Level	A	B	C
1	−40.98	−36.26	−36.29
2	−39.33	−35.40	−35.41
3	−33.18	−36.30	−36.20
4	−33.21	−36.67	−36.16
5	−34.50	−36.57	−37.14
Delta	7.80	1.28	1.73
Ranking	1	3	2

**Table 7:** ANOVA of signal-to-noise ratio.

Source	Free Degree	Seq SS	Adj SS	Adj MS	F	*p*
A	4	267.706	267.706	66.9265	132.39	0.000
B	4	5.066	5.066	1.2664	2.51	0.098
C	4	7.553	7.553	1.8881	3.73	0.034
Residual error	12	6.066	6.066	0.5055	-	-
Total	24	286.391	-	-	-	-

**Table 8:** Model coefficient estimation of signal-to-noise ratio.

Item	Coefficient	Coefficient Standard Error	T	*p*
constant	−36.2391	0.1422	−254.841	0.000
A_1_	−4.7371	0.2844	−16.656	0.000
A_2_	−3.0895	0.2844	−10.863	0.000
A_3_	3.0613	0.2844	10.764	0.000
A_4_	3.0273	0.2844	10.644	0.000
B_1_	−0.0199	0.2844	−0.070	0.945
B_2_	0.8436	0.2844	2.966	0.012
B_3_	−0.0594	0.2844	−0.209	0.838
B_4_	−0.4346	0.2844	−1.528	0.152
C_1_	−0.0508	0.2844	−0.179	0.861
C_2_	0.8323	0.2844	2.926	0.013
C_3_	0.0381	0.2844	0.134	0.896
C_4_	0.0791	0.2844	0.278	0.786
S = 0.7110, R-Sq = 97.9%, R-Sq (adjustment) = 95.8%				

**Table 9:** Experimental design table and results.

Serial Number	C (mm/s)	*h* (µm)	*w* (µm)	*l* (µm)	*d* (µm)
1	10	14.37	3.02	102.86	9.87
2	12	13.23	2.88	99.24	8.23
3	14	12.78	2.98	94.73	8.41
4	16	12.69	2.73	91.63	8.39
5	18	12.34	2.64	88.08	8.17
6	20	11.82	2.32	86.67	8.27

**Table 10:** Measurement results of surface roughness between control group and experimental group.

Position	Control Group			Experimental Group		
	Ra (µm)	Rq (µm)	Rt (µm)	Ra (µm)	Rq (µm)	Rt (µm)
1	1.733	1.087	10.878	1.565	0.949	9.564
2	1.965	1.149	10.564	1.632	1.116	8.954
3	2.032	1.116	10.954	1.884	1.001	11.306
4	1.884	1.201	11.306	1.738	0.867	10.821
5	1.738	0.967	12.021	1.646	0.933	9.933
Mean value	1.870	1.104	11.145	1.693	0.973	10.116
Standard deviation	0.127	0.092	0.503	0.125	0.097	0.893

## Data Availability

The data and code are available from the corresponding author on reasonable request.
